# Development and evaluation of duplex TaqMan real-time PCR assay for detection and differentiation of wide-type and MGF505-2R gene-deleted African swine fever viruses

**DOI:** 10.1186/s12917-020-02639-2

**Published:** 2020-11-09

**Authors:** Zhenhua Guo, Kunpeng Li, Songlin Qiao, Xin-xin Chen, Ruiguang Deng, Gaiping Zhang

**Affiliations:** 1 Key Laboratory of Animal Immunology of the Ministry of Agriculture, Henan Provincial Key Laboratory of Animal Immunology, Henan Academy of Agricultural Sciences, Zhengzhou, People’s Republic of China; 2 ZhengZhou ZhongDao Biotechnology Co., Ltd, Zhengzhou, People’s Republic of China; 3 College of Animal Science and Veterinary Medicine, Henan Agricultural University, Zhengzhou, 450002 People’s Republic of China; 4 Jiangsu Co-innovation Center for Prevention and Control of Important Animal Infectious Diseases and Zoonoses, Yangzhou, People’s Republic of China

**Keywords:** African swine fever virus, Duplex real-time PCR, Differential diagnosis, Gene-deleted strains

## Abstract

**Background:**

African swine fever (ASF) is the most important disease to the pigs and cause serious economic losses to the countries with large-scale swine production. Vaccines are recognized as the most useful tool to prevent and control ASF virus (ASFV) infection. Currently, the MGF505 and MGF360 gene-deleted ASFVs or combined with CD2v deletion were confirmed to be the most promising vaccine candidates. Thus, it is essential to develop a diagnosis method to discriminate wide-type strain from the vaccines used.

**Results:**

In this study, we established a duplex TaqMan real-time PCR based on the B646L gene and MGF505-2R gene. The sequence alignment showed that the targeted regions of primers and probes are highly conserved in the genotype II ASFVs. The duplex real-time assay can specifically detect B646L and MGF505-2R gene single or simultaneously without cross-reaction with other porcine viruses tested. The limit of detection was 5.8 copies and 3.0 copies for the standard plasmids containing B646L and MGF505-2R genes, respectively. Clinical samples were tested in parallel by duplex real-time PCR and a commercial ASFV detection kit. The detection results of these two assays against B646L gene were well consistent.

**Conclusion:**

We successfully developed and evaluated a duplex TaqMan real-time PCR method which can effectively distinguish the wide type and MGF505 gene-deleted ASFVs. It would be a useful tool for the clinical diagnosis and control of ASF.

## Background

African swine fever (ASF), first reported in Kenya in 1921, is a devastating viral disease to the pig industries worldwide [1]. ASF is listed as a “notifiable disease” by the World Organization for Animal Health (OIE) [2]. Especially, its introduction in 2018 to China heralded a new transmission era, as ASF subsequently spread to the entire Southeast Asia in the next year [3–5]. Pig production in China in September 2019 was reduced by 40% compared with that in August 2018, and the price of pork has doubled and even tripled since August 2019 [6]. Currently, the outbreaks of ASF are still ongoing in Africa, the trans-Caucasus region, Eastern Europe, Russian Federation and Asia, which pose a huge challenge to the swine industry in these regions [5]. As there is no effective treatment and vaccines, ASF remains a major concern in both infected and non-infected countries.

The causative agent, ASF virus (ASFV), is the only member of the genus *Asfivirus* within the *Asfarviridae* family [7]. ASFV has a large double-stranded DNA genome with terminal inverted repeats and hairpin loops. The genome size ranged from 170 to 190 kb containing 151–167 genes depending on the strain [1]. Based on the B646L genes, at least 23 genotypes were identified and all of them are present in Africa, whereas only genotypes I and II strains have spread to other continents [5]. The genotype I was historically prevalent in Western Europe and Latin America and was successfully eradicated except Sardinia area in Italy by the mid-1990s [8]. The genotype II is epidemic in the trans-Caucasus region, Eastern Europe and Russian Federation since 2007 and was introduced into Asia in 2018 [9]. Especially in Asia, the outbreaks are going in many countries and appears far from to be effectively controlled [5, 8, 9].

In order to successfully cope with the threat of ASFV, in addition to a very high level of biological safety system, vaccine is the most effective tool to control ASF in the future. Recently, some gene-deleted ASFVs might be potential as live attenuated vaccines [6, 10–13]. Among them, multigene family (MGF) 360-505R and CD2v gene-deleted vaccines, have become one of the most promising ASF vaccine candidates [6, 10]. Thus, it is necessary to develop a diagnosis method to distinguishingwide-type ASFVs infection or gene-deleted vaccine immunization.

In this study, based on the B646L gene and MGF505-2R gene, we developed a novel duplex TaqMan real-time PCR assay to detect and differentiate the wide-type and gene-deleted ASFVs. It will be crucial for the ASFV diagnosis and control with the extensive use of gene-deleted vaccine in the future.

## Results

### Sequence alignment of primers and probes

**Fig. 1:**
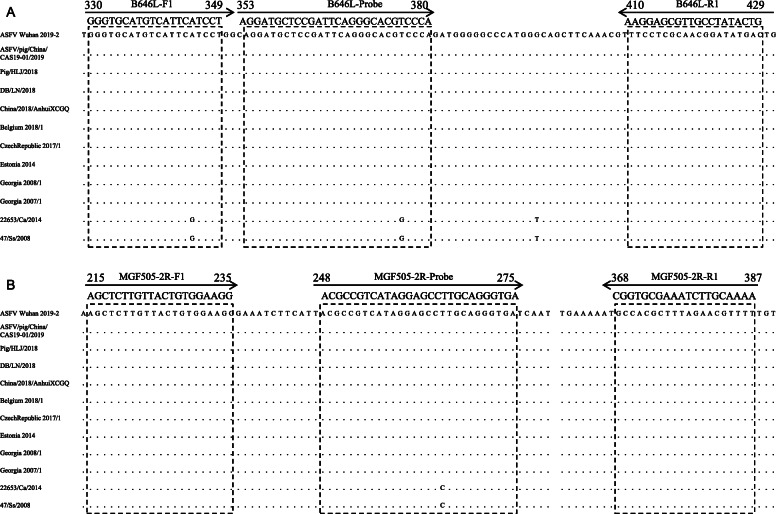
Sequence analysis and alignment of primers and probes in ASFVs. The nucleotides sequence of primers and probes are consistent among the genotype II reference ASFV strains. Compared with the genotype I reference strains, the forward primer and probe targeting B646L gene have one nucleotide mutation at A345G and T376G sites, respectively. The probe targeting MGF505-2R gene has one nucleotide mutation at T265C site

The conservation of these primers and probes were evaluated by clustal *W* method with Megalign program. As shown in Fig. 1, the nucleotides sequence of primers and probes are highly identical among the genotype II reference ASFV strains. Compared with the genotype I reference strains, the forward primer and probe targeting B646L gene have one nucleotide mutation at A345G and T376G sites, respectively. And the probe targeting MGF505-2R gene has one nucleotide mutation at T265C site.

### Analytic specificity assay

**Fig. 2:**
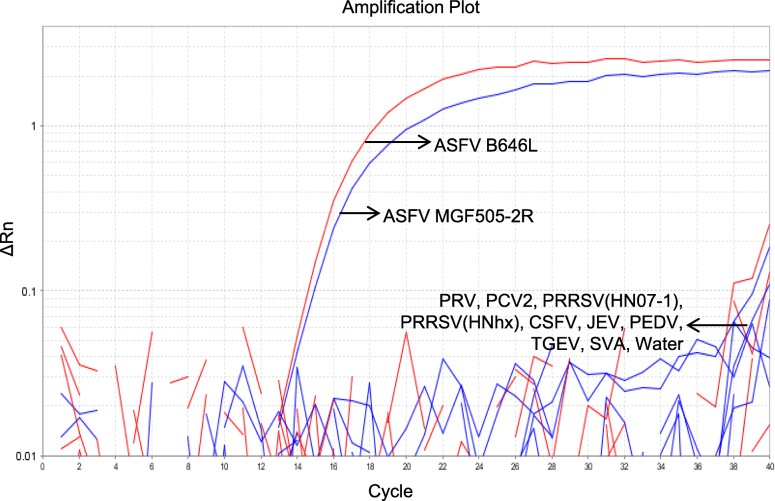
Specificity of the duplex TaqMan real-time PCR assay. Other porcine viruses and the standard recombinant plasmid (pUC57-B646L and pUC57-MGF505-2R) were used to test the specificity

To assess the specificity of the duplex real-time PCR, other DNA/cDNA from PRV, PCV2, PRRSV, CSFV, JEV, PEDV, TGEV and SVA were used as templates. As shown in Fig. 2, amplification curves targeted recombinant plasmids pUC57-B646L and pUC57-MGF505-2R are specific for the fluorescence channels of FAM and VIC, simultaneously. Other samples including the negative control (nuclease-free water) did not show any amplification signals, demonstrating the high specificity of the established duplex real-time PCR.

### Analytic sensitivity assay

**Fig. 3:**
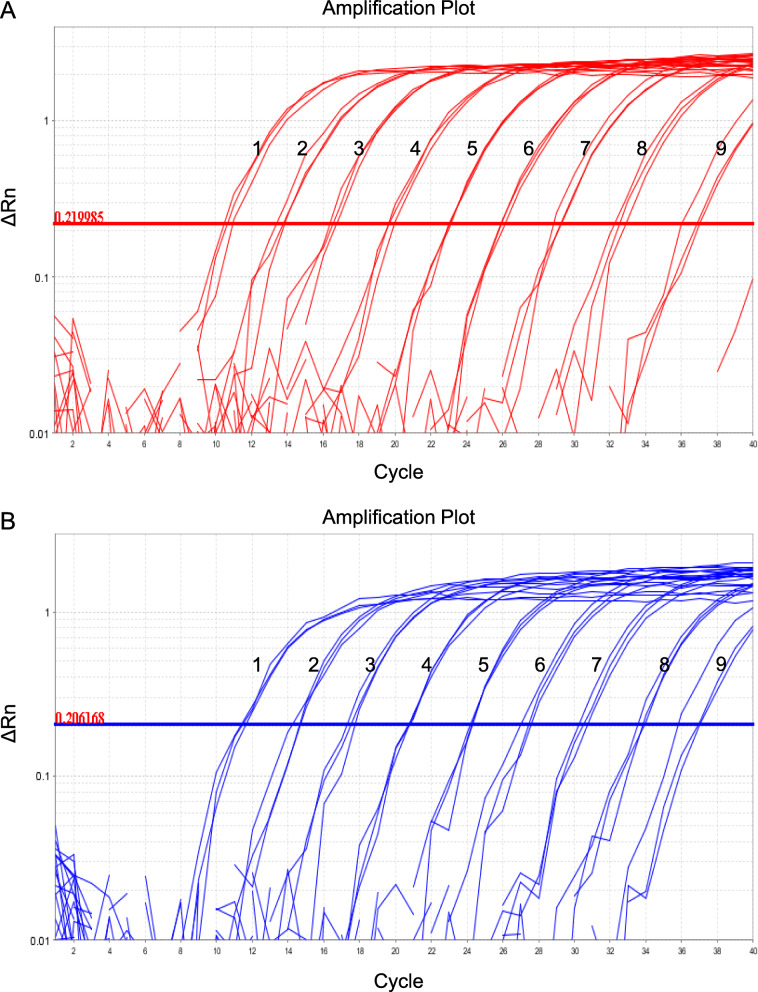
Sensitivity of the duplex real-time PCR assay. Sensitivity of the duplex real-time PCR for B646L gene and MGF505-2R gene. **a** 1–9: 5.8 × 10^8^–10^0^ dilutions of pUC57-B646L plasmid. **b** 1–9: 3.0 × 10^8^–10^0^ dilutions of pUC57-MGF505-2R plasmid

The sensitivity of the duplex real-time PCR was examined using constructed pUC57-B646L and pUC57-MGF505-2R plasmids with serial tenfold dilutions (10^8^ ~ 10^0^ copies/reaction). As shown in Fig. 3, the detection limit for ASFV B646L gene and MGF505-2R gene were 5.0 copies and 3.0 copies, respectively. These data showed that the duplex real-time assay has a good sensitivity.

### Standard curve and repeatability assay

**Fig. 4:**
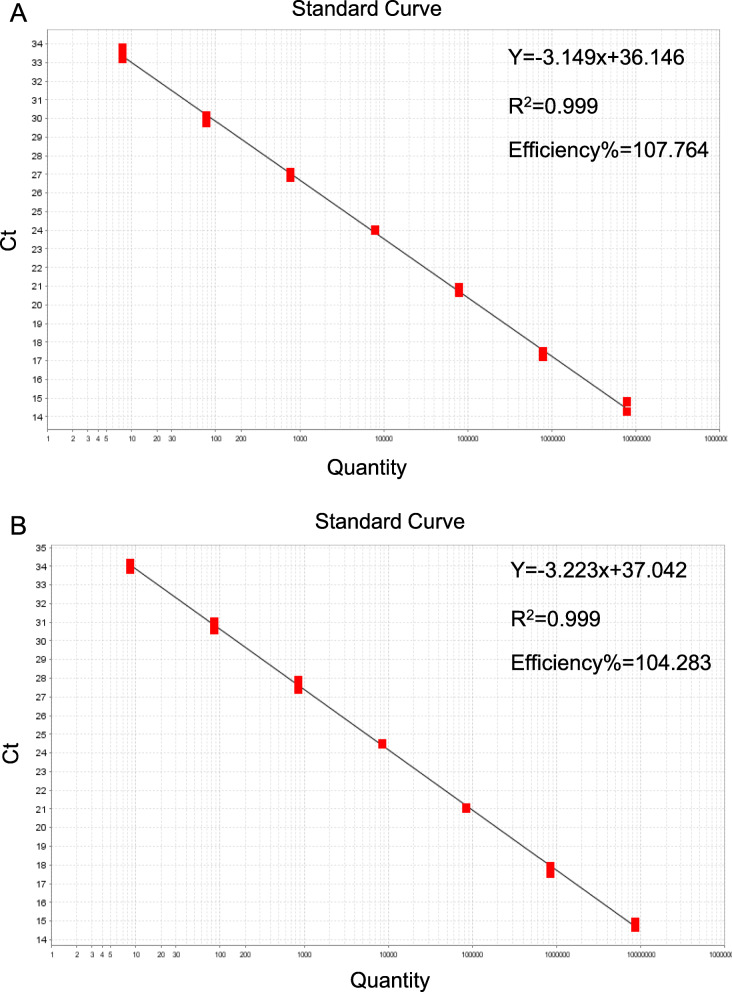
Standard curves for the duplex TaqMan PCR assay. **a** Standard curve for B646L gene, y = − 3.149x + 36.146, *R*^*2*^ = 0.990. **b** Standard curve for MGF505-2R gene, y = − 3.223x + 37.042, *R*^*2*^ = 0.999

Standard curves were generated using 10-fold serial dilutions (10^7^–10^1^) of the recombinant standard plasmids as templates (Fig. 4). Both two standard curves showed a strong linear correlation (*R*^*2*^ = 0.999) between Ct values and quantities of templates. Similar regressions were generated with slopes of − 3.149 and − 3.223, respectively. The repeatability of the assay was further evaluated. As shown in Table 1, the coefficients of variation (CVs) were 0.3–1.7 and 0.1–2.4% in intra-assay and inter-assay, respectively.

**Table 1:** Primers and probes used for the duplex TaqMan Probe-based real-time RT-PCR

Primer/Probe	Sequence (5′-3′)	Amplicon size (bp)
B646L-F1	GGGTGCATGTCATTCATCCT	100
B646L-R1	GTCATATCCGTTGCGAGGAA	
B646L-P1	FAM-AGGATGCTCCGATTCAGGGCACGTCCCA-BHQ1	
MGF505-2R-F1	AGCTCTTGTTACTGTGGAAGG	173
MGF505-2R-R1	AAAACGTTCTAAAGCGTGGC	
MGF505-2R-P1	VIC-ACGCCGTCATAGGAGCCTTGCAGGGTGA-BHQ1	

### Detection of clinical samples

Clinical samples were tested by the established duplex real-time PCR and a commercial ASFV detection kit based B646L gene (Thermo Fisher Scientific) (Table 2). Of the 26 clinical samples, 23 samples were found to be positive by two methods, suggesting a good consistency between the established duplex method and commercial ASFV detection kit when using the B646L gene as targets. Additionally, the VIC signals based on MGF505-2R gene detection were also generated among the 23 positive samples with a similar Ct values with FAM signals, indicating that these positive samples were not MGF360-505R gene deletion strains.

**Table 2:** Reference strains used in this study

No.	Strain name	Accession no.	Regions	Year
1	ASFV/pig/China/CAS19–01/2019	MN172368	China, Hubei	2019
2	ASFV Wuhan 2019–2	MN393477	China, Hubei	2019
3	Pig/HLJ/2018	MK333180	China, Heilongjiang	2018
4	China/2018/AnhuiXCGQ	MK128995	China, Anhui	2018
5	DB/LN/2018	MK333181	China, Liangning	2018
6	Belgium 2018/1	LR536725	Belgium	2018
7	CzechRepublic 2017/1	LR722600	CzechRepublic	2017
8	Estonia 2014	LS478113	Estonia	2014
9	Georgia 2008/1	MH910495	Georgia	2008
10	Georgia 2007/1	NC_044959	Georgia	2007
11	22,653/Ca/2014	MN270980	Italy	2014
12	47/Ss/2008	KX354450	Italy	2008

## Discussion

ASFV has led to tremendous economic losses in the epidemic regions and pose huge challenges to other regions with large scale pig production, including Western Europe, the North America and Latin America [5, 8, 9]. Therefore, huge efforts worldwide have been put on for developing effective and safe ASF vaccines. Recently, several promising attenuated vaccine candidates have been reported using gene deletion strategy. MGF505, MGF360, CD2v, 9GL, DP148R, UK and I177L have been shown to be important virulence determinants of different ASFVs [6, 10–13]. However, the safety and effectivity of most potential vaccine candidates were not fully assessed, and virulence changes induced by gene deletion in one ASFV strain may not be similar in other strains [6]. Deletion MGF505 and MGF360 genes (MGF505-1R, 2R, 3R and MGF360-12 L, 13 L, 14 L) or combined with CD2v did highly attenuated the different ASFV strains and protected pigs against challenge with the virulent parental virus with a satisfactory security [6, 10, 14]. Furthermore, MA-104, a commercially available cell line, was identified to be a suitable substrate for ASFV isolation and growth in vitro, which provide a convenient condition for the large-scale production of attenuated ASFV vaccines [15].

Thus, it is essential to differentiate the wide-type ASFV from vaccines with gene deleted for preventing and controlling ASF. However, most of the existing detection methods, including PCR, real-time PCR, LAMP assay and RPA assay, were developed and improved based on the conserved region of B646L gene, which can not apply to the detection of gene deletions strains [2, 16, 17]. In this study, we developed and evaluated a duplex real-time PCR targeting the B646L gene and MGF505-2R gene. The sequences of primers and probes are highly conserved in the genotype II ASFVs, which are prevalent in Asia, trans-Caucasus region, Eastern Europe and Russian Federation [5, 8, 9]. The generation of two signals (FAM and VIC) indicated the infection of wide type ASFVs. While a single FAM signal suggested the existence of MGF505 gene-deleted ASFVs.

Our data also showed that the duplex TaqMan PCR assay can accurately detect the ASFVs while other swine viruses, including PRV, PCV2, PRRSV, CSFV, JEV, PEDV, TGEV and SVA, gave negative results. The sensitivity assay demonstrated that this method can detect 5.0 copies and 3.0 copies of pUC57-B646L and pUC57-MGF505-2R standard plasmid DNA, respectively. The results of the standard curve showed a strong linear correlation coefficient, a stable repeatability and good amplification efficiency. For clinical samples testing, the results using our assay were well consistent with the commercial ASFV detection kit (Thermo Fisher Scientific) when the Ct value is lower than 35. However, it is not well comparable when the Ct value is greater than 35. It makes sense that in most PCRs the higher the Ct value, the lower the repeatability when Ct value is greater than 36 [18].

## Conclusion

In conclusion, we established a duplex real-time PCR assay based on the B646L gene and MGF505-2R gene, which can successfully distinguish the wide type and MGF505 gene-deleted ASFVs. The duplex method was proved to be specific, sensitive and reliable for clinical samples. This newly developed assay represents a useful tool for the clinical diagnosis of ASFV infections.

## Methods

### Virus strains

Pseudorabies virus (PRV) strain HeNLH/2017 (Accession no. MT775883), Porcine reproductive and respiratory syndrome virus (PRRSV) strain HN07–1(Accession no. KX766378) and HNhx (Accession no. KX766379), Senecavirus A strain HeNNY-1/2018 (Accession no. MK357116) and porcine circovirus 2 (PCV2) strain DF-1 (Accession no. JN119255) were isolated and stored in our lab. Classical swine fever live vaccines (CSFV, strain CVCC AV1412), Porcine transmissible gastroenteritis (TGEV, strain WH-1R) and Porcine epidemic diarrhea live vaccine (PEDV, strain AJ1102-R) and Swine Japanese encephalitis live vaccine (strain SA14–14-2) were purchased from Wuhan Keqian Biology Co., Ltd. and stored in our lab.

### Viral nucleic acid extraction

Viral nucleic acid was extracted from each sample using TaKaRa MiniBEST Viral RNA/DNA Extraction Kit Ver.5.0 according to the manufacturer’s instructions. The cDNA was synthesized using the PrimeScript™RT Master Mix Kit (TaKaRa Biotechnology Co., Ltd., Dalian, China). The viral DNA/cDNA were stored at − 40 °C for further research.

### Primers and probes design

The primers and probes were designed and evaluated by Primer3Plus (http://www.primer3plus.com) based on the genome sequence of Pig/HLJ/2018 (Accession no. MK333180) (Table 3). The probe for B646L gene was labeled with reporter dye 6-carboxyfluorescein (FAM) and the 3′-quencher BHQ1. The probe for MGF505-2R gene was labeled with reporter dye VIC and the 3′-quencher BHQ1. The sequence alignment was analyzed by clustal *W* method with Megalign program (DNASTAR, Inc., Madison, WI, USA) to confirm the conservation of primers and probes among the genotype II and I reference ASFV strains (Table 4) (Fig. 1). The primers and probes above were all synthesized by Sangon Biotech, China.

**Table 3:** Repeatability of duplex TaqMan real-time PCR

Plasmid	Copy number	Inter-coefficient of variation			Intra-coefficient of variation		
		Ct mean	Ct SD	CV%	Ct mean	Ct SD	CV%
pUC57- B646L	5.8 × 10^7^	13.50	0.23	1.7	13.19	0.29	2.2
	5.8 × 10^6^	16.79	0.29	1.7	16.56	0.35	2.1
	5.8 × 10^5^	19.53	0.12	0.6	19.57	0.03	0.1
	5.8 × 10^4^	22.64	0.08	0.3	22.35	0.24	1.1
	5.8 × 10^3^	26.02	0.14	0.5	25.99	0.12	0.5
	5.8 × 10^2^	28.31	0.27	1	28.81	0.38	1.3
	5.8 × 10^1^	31.75	0.08	0.3	32.05	0.27	0.8
pUC57- MGF505-2R	3.0 × 10^7^	12.62	0.14	1.1	12.62	0.23	1.9
	3.0 × 10^6^	15.61	0.21	1.3	15.79	0.35	2.2
	3.0 × 10^5^	19.02	0.08	0.4	18.94	0.38	2
	3.0 × 10^4^	21.85	0.26	1.2	21.78	0.18	0.8
	3.0 × 10^3^	25.82	0.22	0.9	25.60	0.37	1.5
	3.0 × 10^2^	28.46	0.21	0.7	28.51	0.54	1.9
	3.0 × 10^1^	31.43	0.16	0.5	31.70	0.76	2.4

**Table 4:** Detection of clinical samples using the established duplex real-time PCR assay

Sample no.	Duplex TaqMan PCR assay (Ct value)	VetMAX™ ASFV Detection Kit (Ct value)	Sample no.	Duplex TaqMan PCR assay (Ct value)	VetMAX™ ASFV Detection Kit (Ct value)
1	27.27/27.81	27.41/−	14	34.19/34.64	35.74/−
2	19.58/19.44	20.7/−	15	31.05/29.86	31.98/−
3	17.87/17.42	18.4/−	16	33.45/33.85	33.9/−
4	18.97/18.4	19.42/−	17	32.91/31.9	32.77/−
5	14.32/13.02	15.24/−	18	31.97/31.84	32.99/−
6	16.21/14.87	16.38/−	19	30.97/30.12	31.94/−
7	16.87/17.46	17.61/−	20	34.49/33.73	36.52/−
8	27.66/27.11	27.72/−	21	33.99/35.61	35.64/−
9	26.37/26.51	26.72/−	22	35.62/37.0	35.19/−
10	19.05/18.56	19.81/−	23	34.51/35.51	35.42/−
11	18.23/17.49	18.78/−	24	37.16/39.76	36.99/−
12	31.68/30.82	31.85/−	25	36.12/38.4	undetermined
13	34.67/34.3	32.65/−	26	36.95/−	undetermined

Note: “-” means no detection, “undetermined” means no signal。

### Recombinant standard plasmid construction

The full length of B646L gene (1941 base pairs) and MGF505-2R gene (1581 base pairs) of ASFV genome (Accession no. MK333180) were synthetized and cloned into pUC57 vector by Sangon (Shanghai, China), respectively. The concentration of these recombinant standard plasmids was determined by NanoDrop One (ThermoFisher Scientific). According to the DNA copy number calculation formula [double strand DNA copy numbers (copies/mL) = 6.02 × 10^23^ (copies/mL) × concentration (g/mL) / DNA length× 660], the copy numbers of pUC57-B646L and pUC57-MGF505-2R plasmids were 2.9 × 10^9^ copies/μL and 1.5 × 10^9^ copies/μL, respectively. All these plasmids were stored in − 20 °C before use.

### Analytic specificity determination

The specificity of the duplex TaqMan real-time PCR assay was first evaluated using combined control plasmids and 9 other viral DNA/cDNA, including PRV, PCV2, PRRSV (HN07–1 and HNhx), CSFV, JEV, PEDV, TGEV and SVA. The nuclease-free water was used to be negative control. The reaction volume for real-time PCR was 25 μL which contained 2 μL template (standard plasmids or viral DNA/cDNA), 2 μL primers mixture (2.5 μM for each primer) and 1 μL probes mixture (5 μM for each probe) (the final optimized concentrations of the primers and probes were 0.2 μM), 0.25 μL ROX Reference Dye II (50x), 12.5 μL Premix Ex Taq™ (Probe qPCR, 2 x Conc.) and then add nuclease-free water to 25 μL. The amplification was carried out under the default programs using 7500 Fast Real-Time PCR System. The reaction program was pre-denaturation 95 °C for 20s, and then 40 cycles of 95 °C for 3 s, 60 °C for 30s.

### Analytic sensitivity and repeatability determination

The standard plasmids were serially diluted 10-fold with nuclease-free water, corresponding to 5.8 × 10^9^ to 5.8 × 10^0^ copies/reaction for pUC57-B646L and 3.0 × 10^9^ to 3.0 × 10^0^ copies/reaction for pUC57-MGF505-2R, respectively. Standard curve and repeatability were determined from the triplicates of each dilution. The reaction system and amplification condition referred to the methods described above.

### Clinical sample detection

A total of 26 pig serums collected from suspected ASFV infected pigs were detected by our novel duplex TaqMan real-time PCR method and VetMAX™ ASFV Detection Kit (Thermo Fisher Scientific). The serums and extracted DNA were provided and stored by ZhengZhou ZhongDao Biotechnology Co., Ltd. For the reaction system, the amount of template (DNA) was 5 μL and the other components were added as above.

## Data Availability

The material analyzed during the current study are available from the corresponding author on reasonable request.

## Abbreviations

ASF: African swine fever
ASFV: ASF virus
MGF: Multigene family
PCR: Polymerase chain reaction
Ct: Cycle threshold
CV: Coefficients of variation
LAMP: Loop-mediated isothermal amplification
RPA: Recombinase polymerase amplification
PRV: Pseudorabies virus
PCV2: Porcine circovirus
PRRSV: Porcine reproductive and respiratory syndrome virus
CSFV: Classical swine fever virus
JEV: Japanese encephalitis virus
PEDV: Porcine epidemic diarrhea live vaccine
TGEV: Transmissible gastroenteritis virus
SVA: Senecavirus A
